# Novel Molecules in Diabetes Mellitus, Dyslipidemia and Cardiovascular Disease

**DOI:** 10.3390/ijms24044029

**Published:** 2023-02-17

**Authors:** Cosmin Mihai Vesa, Simona Gabriela Bungau

**Affiliations:** 1 Department of Preclinical Disciplines, Faculty of Medicine and Pharmacy, University of Oradea, 410073 Oradea, Romania; 2 Department of Pharmacy, Faculty of Medicine and Pharmacy, University of Oradea, 410087 Oradea, Romania

The purpose of this Special Issue is to present the impact in clinical practice as well as in medical research of novel molecules that have been introduced in the treatment of diabetes mellitus, dyslipidaemia, and cardiovascular disease. The topic focuses on the improvements in disease outcomes as well as the numerous beneficial effects of the use of certain medications such as glucagon-like peptide 1 (GLP-1) agonists, glucose-dependent insulinotropic polypeptide (GIP) agonists, and dual GLP-1/GIP agonists for the treatment of diabetes mellitus and obesity, as well as medication such as sacubitril/valsartan for the treatment of heart failure. Included in the topic is also the presentation of certain novel molecules such as derivatives of quinopimaric acid, useful in the inhibition of some enzymes involved in the glucose metabolism, or what is also presented is novel applications of the administration of older molecules (such as nicotinamide) for the inhibition of the key pathogenetic processes involved in diabetes complications. We believe that the number of real-life studies and in vivo and in vitro research of the effects of these drugs is, to date, rather low, leading to a lack of understanding of the complex molecular effects and pleiotropic effects of these medications, as well as incomplete knowledge regarding the repurposing of these drugs in clinical practice. Thus, this Special Issue is incredibly topical and important, and we look forward to your submissions which will help to clarify the issues surrounding such drugs.

Teryakova’s team carried out a very interesting study where abietane-type derivatives with arylidene, heterocyclic, nitrile, and acetylene fragments were used to inhibit the enzyme, α-Glucosidase (α-GLy); the efficacy of these novel compounds was investigated in vitro and by molecular modelling approaches. The complex investigation, that included absorption, distribution, metabolism, excretion, and toxicity (ADMET) profiling or kinetic studies of the novel synthesized compounds, led to the conclusion that a lead diterpene indole with an alkyne substituent of 45 was identified as a competitive inhibitor for the enzyme α-GLy involved in the glucose metabolism, which provides an insight into novel antidiabetic drug development [1].

The team led by Zlacká demonstrated that phosphofructokinase 15 (PFK15), a synthetic compound, can act together with sunitinib and inhibit glycolysis in human umbilical vein endothelial (HUVEC) cells, therefore reducing angiogenesis, an important pathophysiological process in the apparition and progression of diabetic complications [2].

Lee’s team had an important contribution with two impressive articles. In one study, they demonstrated that nicotinamide mononucleotide intraperitoneal injections in mice prevented retinal ischemia/reperfusion (I/R) injury, a mechanism that was explained by the activation of certain antioxidant pathways [3], while in the other study, the administration of nicotinamide mononucleotide in mice, where a surgical unilateral common carotid artery occlusion was performed, was associated with a reduced retinal dysfunction, preserved nicotinamide adenine dinucleotide (NAD^+^) levels, normal glycolysis, and increased antioxidant activity mechanisms which can be important in clinical practice for the amelioration of diabetic retinopathy [4].

With respect to the above-mentioned data, Iovanovici et al. analysed the beneficial role of sacubitril/valsartan therapy in patients with heart failure with a reduced ejection fraction (HFrEF) and patients with heart failure with a preserved ejection fraction (HFpEF). The review presented the main clinical trials results, where it was demonstrated that the utilisation of sacubitril/valsartan therapy in HFrEF patients reduced the rehospitalization rate due to heart failure and improved patients’ symptoms, while other trials demonstrated reversible cardiac remodelling and the drop of N-terminal proBNP (NT-proBNP) levels when the angiotensin receptor–neprilysin inhibitor (ARNI) is administered [5]. In HFpEF, sacubitril/valsartan was associated with reduced NT-proBNP levels, although there were not any significant results in cardiovascular mortality, the mortality of all causes, or an improvement in the NYHA class [5].

Another interesting review was provided by Forzano I. et. which analysed the benefits of a novel medication, tirzepatide, a molecule that acts by the dual agonism of GLP-1/GIP receptors. Tirezepatide’s efficacy in reducing blood glucose, weight, and blood pressure values was confirmed in five clinical trials in patients with type 2 diabetes mellitus, while in non-diabetic obese patients, a weekly administration of the drug, in various concentrations, produced remarkable results in reducing body weight [6].

Fiorentino et al. analysed the benefits of statin use in children, a population where this medication is rarely used, and demonstrated numerous benefits such as a reduction in the atherosclerosis burden, with fewer side effects than in adults because of the presence of a very low number of comorbidities [7].

Numerous molecular pathways, such as those activated by GLP-1 agonists [8], still need to be investigated for reducing the impact that diabetes mellitus and cardiovascular disease have on human health, especially given the fact that the molecular understanding of a disease creates the possibility of targeted therapies and personalized care.

## References

[1] Tretyakova E., Smirnova I., Kazakova O., Nguyen H.T.T., Shevchenko A., Sokolova E., Babkov D., Spasov A. (2022). New Molecules of Diterpene Origin with Inhibitory Properties toward α-Glucosidase. Int. J. Mol. Sci..

[2] Zlacká J., Murár M., Addová G., Moravčík R., Boháč A., Zeman M. (2022). Synthesis of Glycolysis Inhibitor PFK15 and Its Synergistic Action with an Approved Multikinase Antiangiogenic Drug on Human Endothelial Cell Migration and Proliferation. Int. J. Mol. Sci..

[3] Lee D., Tomita Y., Miwa Y., Shinojima A., Ban N., Yamaguchi S., Nishioka K., Negishi K., Yoshino J., Kurihara T. (2022). Nicotinamide Mononucleotide Prevents Retinal Dysfunction in a Mouse Model of Retinal Ischemia/Reperfusion Injury. Int. J. Mol. Sci..

[4] Lee D., Tomita Y., Miwa Y., Jeong H., Shinojima A., Ban N., Yamaguchi S., Nishioka K., Negishi K., Yoshino J. (2022). Nicotinamide Mononucleotide Protects against Retinal Dysfunction in a Murine Model of Carotid Artery Occlusion. Int. J. Mol. Sci..

[5] Iovanovici D.-C., Bungau S.G., Vesa C.M., Moisi M., Babes E.E., Tit D.M., Horvath T., Behl T., Rus M. (2022). Reviewing the Modern Therapeutical Options and the Outcomes of Sacubitril/Valsartan in Heart Failure. Int. J. Mol. Sci..

[6] Forzano I., Varzideh F., Avvisato R., Jankauskas S.S., Mone P., Santulli G. (2022). Tirzepatide: A Systematic Update. Int. J. Mol. Sci..

[7] Fiorentino R., Chiarelli F. (2023). Statins in Children, an Update. Int. J. Mol. Sci..

[8] Iorga R.A., Bacalbașa N., Carsote M., Bratu O.G., Stănescu A.M.A., Bungău S., Pantiș C., Diaconu C.C. (2020). Metabolic and cardiovascular benefits of GLP-1 agonists, besides the hypoglycemic effect (Review). Exp. Ther. Med..

